# A Hint from Socialism

**Published:** 1913-12

**Authors:** 


### A Hint From Socialism

A socialistic paper has recently given prominence to a scheme for bringing capital to terms by cornering the money of the country. Workingmen are urged not to deposit their money in banks, from which it may be loaned to capital or otherwise used in business, and not to pay any bills which they can postpone. Just how labor is to "get out from under" the anticipated and logical crash of industry is not explained. We suggest that medical workingmen who do not, on the average, earn plumber's wages, and who do not have an eight-hour day, bear in mind the second point suggested.

